# Comparison of neuromuscular and cardiovascular exercise intensity and enjoyment between standard of care, off-the-shelf and custom active video games for promotion of physical activity of persons post-stroke

**DOI:** 10.1186/s12984-021-00850-2

**Published:** 2021-04-14

**Authors:** Judith E. Deutsch, Aurora James-Palmer, Harish Damodaran, Urska Puh

**Affiliations:** 1 Rivers Lab, Department of Rehabilitation and Movement Science, Rutgers School of Health Professions, 65 Bergen Street, Newark, NJ 07101 USA; 2 Motor Behavior Lab, Department of Rehab and Movement Science, Rutgers School of Health Professions, 65 Bergen Street, Newark, NJ 07101 USA; 3 Department of Physiotherapy, Faculty of Health Sciences, University of Ljubljana, Ljubljana, Slovenia

**Keywords:** Active video games, Exergaming, Virtual reality, Stroke, Kinect

## Abstract

**Background:**

Active video games have been embraced for the rehabilitation of mobility and promotion of physical activity for persons post-stroke. This study seeks to compare carefully matched standard of care stepping activities, off-the-shelf (non-custom) active video games and custom active video games that are either self-paced or game-paced for promoting neuromuscular intensity and accuracy, cardiovascular intensity, enjoyment and perceived effort.

**Methods:**

Fifteen persons (ages 38–72) with mild to moderate severity in the chronic phase post-stroke (average 8 years) participated in a single group counter balanced repeated measures study. Participants were included if they were greater than 6 months post-stroke, who could walk 100 feet without assistance and stand unsupported for three continuous minutes. They were excluded if they had cardiac, musculoskeletal or neurologic conditions that could interfere with repeated stepping and follow instructions. In a single session located in a laboratory setting, participants executed for 8.5 min each: repeated stepping, the Kinect-light race game, two custom stepping games for the Kinect, one was repeated and self-paced and the other was random and game paced. Custom video games were adjusted to the participants stepping volume. Ten-minute rest periods followed the exercise during which time participants rested and completed the PACES an enjoyment questionnaire. Participants were instrumented with a metabolic cart and heart rate sensor for collection of cardiovascular intensity (METs and % of max HR) data. Stepping frequency, accuracy and pattern were acquired via video. Data were analyzed using a RMANOVA and post-hoc comparison with a Holm's/Sidak correction.

**Results:**

Neuromuscular intensity (repetitions) was significantly greater for the off-the-shelf and self-paced custom game, however accuracy was greater for the custom games. Cardiovascular intensity for all activities took place in the moderate intensity exercise band. Enjoyment (measured with a questionnaire and rankings) was greater for the custom active video games and rate of perceived exertion was lower for the custom active video games.

**Conclusions:**

Custom active video games provided comparable intensity but better accuracy, greater enjoyment and less perceived exertion than standard of care stepping activities and a carefully matched off-the-shelf (non-custom) video game. There were no differences between the game-paced and self-paced custom active video games.

*Trial registration:* NCT04538326.

## Background

Activity and participation are often limited for persons-post stroke. Mobility problems are reported by 58% of people in chronic stage after stroke, fatigue in 52%, and falls in 44% [1]. Low cardiorespiratory fitness and other impairments, such as decreased balance and muscle strength interact to drive post-stroke activity limitations and participation restriction [2]. People post-stroke may only manage to walk short distances, walk slowly, fatigue more often during everyday activities and are at greater risk for falls. Their sedentary lifestyle is related not only to decreased walking ability, but it also increases risk for another stroke and other cardio-vascular diseases, depression, psychosocial disfunctions and decreased quality of life [3]. Physical exercise programmes may serve to increase physical fitness and mobility of people post-stroke [2, 4].

Active video games (AVGs) became available for movement focused rehabilitation when gaming consoles such as the Play Station II (PS2) with the Eye Toy camera, the Nintendo Wii and the Microsoft Xbox with the Kinect camera were trialed by clinicians and tested by researchers [5–7]. Analyses of the non-custom AVGs associated with the consoles identified elements of motor learning and therapeutic exercise in order to guide the application of AVGs into physical therapy practice, as well as rehabilitation research [8–11]. Recent surveys of virtual reality and AVGs confirm that AVGs have become part of the repertoire of rehabilitation tools used for persons post-stroke. Physical and occupational therapists, surveyed about virtual reality and video games in both in Canada and the United States, report primarily using non-custom AVGs for commercial consoles such as the Nintendo Wii and the Microsoft Kinect to treat balance and fitness for persons post-stroke. [12] Boyne and colleagues [13] reported AVGs were used to promote aerobic exercise for persons post-stroke in a variety of clinical settings. AVGs appear to meet the need for rehabilitation of mobility and fitness deficits experienced by persons post-stroke.

Evidence to support these non-custom AVGs has been increasing with their potential application for rehabilitation of upper limb use, [14] balance and mobility [15] as well as promotion of physical activity (PA) [16, 17] for persons post-stroke. There has also been an interest in developing active custom games for upper limb use [18] and balance [19–21]. A rationale often proposed for incorporating AVGs into stroke rehabilitation is their potential to increase participant motivation. Several elements of AVGs may account for increasing or be associated with motivation; these include game mechanics (such as scoring), performance feedback, “the right level of challenge” and enjoyment [22]. In persons post-stroke in an inpatient setting who played upper limb games using a manipulandum to control a game displayed on a tv, the use of feedback was shown to be superior in improving movement speed and smoothness to games that did not provide performance feedback. Importantly, they also had greater motivation (measured with the interest and enjoyment subscale of the Intrinsic Motivation Inventory) with the game play [23]. Game mechanics that involved scoring and “operant conditioning” both yielded higher performance metrics when playing an upper limb labyrinth game with those features [24].

Enjoyment is proposed as an important construct to recommend AVG play [25]. It has been measured by the same group of investigators while persons post-stroke: (1) interacted with the PS2 Eye Toy, [5] (2) compared enjoyment while playing the PS2 and the Wii [26] and (3) played Kinect Sports and Adventure games [11]. Across all three studies, enjoyment was reported by persons post-stroke. Enjoyment was also identified as an important construct linking engagement for motor learning in virtual environments [27]. It is speculated that enjoyment is a factor that also promotes motivation and may lead to sustained and intense activity required for neural, musculoskeletal and cardiovascular plasticity required for stroke rehabilitation. In fact, several groups have shown that persons post-stroke exercising in virtual environments and AVGs achieve high levels of repetitions [28] and a higher number of repetitions (a measure of intensity) compared to standard therapy [29].

While the non-custom AVGs have shown promise and they are appealing because of their low-cost, high quality graphics and variety, they have several important limitations for incorporating them into therapy. Often, they are not user-centered, do not meet therapeutic goals, nor provide performance metrics, nor promote desirable movement patterns [30, 31]. One of the consistent limitations, of these off-the-shelf consoles and their games, reported by both clinicians and persons post-stroke, is the lack of control in adjusting exercise parameters such as speed or difficulty [32]. The lack of game control is especially true for game-paced, rather than player or self-paced games. AVGs and virtual environments may also not reliably promote optimal movement patterns [33–35]. This is particularly important for persons post-stroke who exhibit asymmetrical movements. The promise that some of these limitations could be addressed with games that were specifically designed for rehabilitation was heralded with the open development kit of the Kinect (STK). The combination of markerless motion capture with customized software could overcome some of the limitations of non-custom off-the shelf games as well the cost of more specialized systems. Several groups have developed custom games with the Kinect primarily for balance that allow customization and clinician control and have been shown to efficacious in rehabilitation post stroke [19] or superior to off -the-shelf analogs in older adults [36]. There are however, to our knowledge no studies that carefully compare movement performance, energetics and enjoyment of off-the-shelf video games with custom games as well as standard of care activities for persons with stroke.

Therefore, in this study we sought to compare if carefully matched standard of care activities and off-the-shelf AVG with customized AVGs produced a desirable exercise intensity and player experience for persons post-stroke. The specific purpose of this study was to determine if: (1) Movement outcomes of neuromuscular intensity (repetitions) and movement accuracy were superior when playing an off-the-shelf AVG, compared to custom AVGs or standard of care therapy, (2) Cardiovascular intensity outcomes of (METs and % of HR_max_) were superior when playing an off-the-shelf AVGs, compared to custom AVGs or standard of care therapy, (3) enjoyment, game preference and perceived effort differed when playing an off-the-shelf AVG, compared to custom AVGs or standard of care therapy and (4) there was a difference in neuromuscular and cardiovascular intensity as well as enjoyment and game preference between the two customized AVGs that are self-paced compared to game-paced. We hypothesized that neuromuscular and cardiovascular intensity, would differ between custom AVGs, off-the shelf AVGs and standard of care; and that enjoyment would be greater and perceived effort lower for the custom AVGs compared to the off-the-shelf AVG and standard of care.

## Methods

**Fig. 1:**
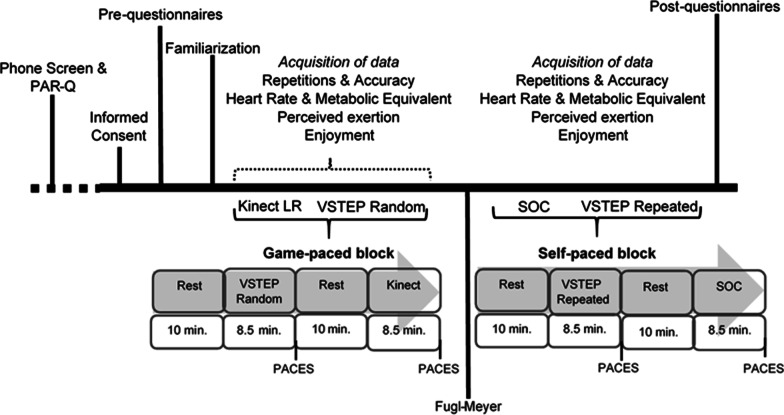
The study protocol. The two blocks of the study conditions: kinect light race (Kinect-LR) and VSTEP random, and standard of care (SOC) and VSTEP repeated are counterbalanced for each subject; *PAR-Q* Activity Readiness Questionnaire, *PACES* Physical Activity Enjoyment Scale

This study was a single group repeated measures mixed methods design approved by the Rutgers Institutional Review board. Data were acquired in a single session with all study activities listed in Fig. 1. A sample of convenience was recruited within the community of Newark, NJ and surrounding areas through flyers and word of mouth. Participants of previously completed studies conducted by this laboratory were also informed of this study opportunity.

### Participants

Individuals in the chronic phase post-stroke were eligible to participate if they walked 100 feet without assistance and could stand unsupported for three continuous minutes. Individuals were excluded if they had a history of any of the following: (1) severe heart disease, (2) heart attack, (3) valve replacement or coronary artery bypass surgery, (4) severe lung disease, (5) uncontrolled diabetes, (6) traumatic brain injury or neurological disorder other than stroke, (7) an unstable medical condition or musculoskeletal disorder such as arthritis or hip and knee surgery, or (8) any other condition that would interfere with repeated stepping. Interested individuals engaged in a phone screen and completed the Activity Readiness Questionnaire (PAR-Q), [37] receiving clearance from their primary care physicians when applicable.

### Study protocol

After consenting, demographic data including PA and video game history were collected at the start of the session. Participants were familiarized with the four exercise conditions which were divided into two blocks, a game-paced block and a self-paced block. Familiarization included physical practice and reporting exertion with the Borg Rating of Perceived Exertion Scale (RPE) [38, 39]. Participants were allowed to use their assistive devices during familiarization and data collection and were guarded as appropriate for safety. While participants practiced the custom game, the VSTEP, in both the repeated and random conditions, the volume of stepping for each game was calibrated independently for the affected and unaffected lower extremities. Calibration of the custom game was completed and understanding of game play for each condition was confirmed before data collection commenced. Between the two activity blocks, participants were tested for their motor control using the lower extremity section of the Fugl Meyer Assessment Scale (scored between 0 and 34 points). [40].

Participants were instrumented with a heart rate monitor (*Polar HC-10, Finland)* and a mask for the portable metabolic cart unit (*Cosmed KB4*) to obtain physiological data. Resting physiological data were obtained while participants were comfortably seated in a dimly lit environment during ten-minute rest periods preceding the performance of each condition. Then, during each condition physiological data were continuously obtained. Event markers were recorded on the metabolic unit to bookend each rest period and each study condition to aid in data extraction. The RPE was recorded at mid-point and end of each condition. A study team member held up a clipboard with the numbers 6–20 and their respective perceived exertion levels (rest—maximum exertion) written in bold print [38, 39]. Participants pointed to or verbally indicated their RPE score when asked.

The four exercise conditions, as illustrated in Fig. 1, were selected to compare an off-the-shelf video game, a custom video game (in two modes), and a standard of care condition. They were performed for 8.5 min with bouts of stepping gameplay interleaved with 30-s bouts of marching. The duration of the conditions was selected for participants to reach an exercise plateau, in order to extract and analyze metabolic data and derive METs [17]. The four conditions were selected to be equivalent. For the self-paced block, the movements were identical in duration and movement direction. For the game-paced block, the pattern of stepping was restricted to the forward, lateral and diagonal directions, not allowing for backward stepping. Participants were instructed to “keep moving” for the entire 8.5 min in all conditions. The off-the-shelf game only allowed for 1.5 min of gameplay in one bout and required 30 s to restart the game. The marching bouts were added to address this constraint of the off-the-shelf game and the other conditions were designed to include marching activities to ensure equivalence of the conditions. The visual display of all three video games was rendered with a short throw projector with a size of 5 ft high by 4 ft wide. The standard of care activity did not have a visual display. For a comparison of characteristics of the four study conditions see, Table 1. Each condition is described as follows:

**Table 1:** The four study conditions and their characteristics

Characteristics	Game-paced block		Self-paced block	
	Kinect-LR	VSTEP random	VSTEP repeated	SOC
Visual display		Two displays used for both VSTEP conditions		White wall
Visual and audio feedback	Stepping only	Stepping and marching	Stepping and marching	None
VR perspective	Third	First	First	NA
Color differentiation of lower extremities	No	Yes	Yes	NA
Stepping volume (distance)	Game-determined	Customized	Customized	Self-determined
Stepping pattern	Random	Random	Repeated	Repeated
Stepping pace	Game-determined	Game-determined	Self-determined	Self-determined

*SOC* standard of care, *LR *light race, *VR* virtual reality

Game-Paced Block

1. Kinect Light Race (Kinect-LR): Participants played the off-the-shelf X-Box Kinect game Light Race at “easy” difficulty. For this game, there was a full body avatar and circle of four tiles (1) right side, (2) right front, (3) left front, and 4) left side. The size of the circle and the position of the tiles was the same for all participants. Participants were instructed to step on the tiles when they changed color. Participants were instructed to march between game play bouts.
2. VSTEP Random (VRAN): Participants played the custom VSTEP game in the random mode. The VSTEP was originally developed and validated by the authors [41]. The current version was developed using UNITY 3d game engine (*Unity Technologies, Denmark*) and C# programing language and uses the Kinect 1 sensor (*Microsoft Corp, United States*) for skeletal tracking of the lower extremities. For this game, there were avatar shoes representing the lower extremities, a center square and 6 tiles (1) right side, (2) right diagonal, (3) right front, (4) left front, (5) left diagonal, (6) left side. In the random mode participants were directed to step on blue tiles with their left foot and red tiles with their right foot. The speed and location of the tiles was directed by the game. In between each stepping bout participants were directed to lift their feet in a march-like step to contact an onscreen ball.
  Self-Paced Block
3. VSTEP Repeated (VREP): Used the VSTEP with the same onscreen display, as VSTEP Random, with the avatar shoes, the center square, and onscreen tiles. In this condition, however, participants played the VSTEP in the repeated mode followed a specific sequence of stepping and marching in place for 30-s until a new direction was stated. Visual feedback and coinciding auditory feedback were provided as participants observed their foot avatar from a first person view and the target and center tiles alternated changing color at the pace set by the participant’s stepping. Each lower extremity had its own color. The stepping sequence was left side step, right side step, left forward step, march, right diagonal step, left diagonal step, right forward step, march, left side step, rights side step, left forward step, right diagonal step, march, right forward step, left side step, right side step, and march. This condition included the same march-like gameplay activity with the onscreen ball as described above.
4. Standard of Care (SOC): The identical set of movements in the same sequence as the VREP condition were performed for the SOC condition, but without any visual targets or feedback.

Participants completed the Physical Activity Enjoyment Scale (PACES) immediately after each activity, for a total of four times. The PACES is an 18-item scale that assesses enjoyment for PA by asking patients to rate how they feel at the time of PA on a 7-point Likert scale, from 1 (I enjoy it) to 7 (I hate it). Scores range between 18 and 126, with higher scores indicating higher enjoyment [42, 43]. After completing all four conditions, participants ranked ordered their preferences based on what they liked best. They also ranked each condition based on difficulty from easiest to hardest and answered open-ended questions about their experiences of each condition. Open ended questions were posed as follows: “can tell us what you experienced and why you selected item X as the one you liked the most” and “Is there anything that you would like to tell us that we did not ask?” Participants were encouraged to elaborate on their responses and the tester paraphrased the response to get confirmation that it was understood clearly.

### Data extraction and reduction

#### Neuromuscular intensity and performance: repetitions and accuracy

Each study condition was video recorded with an iPAD from behind the participant in order to capture both the display screen and the participant. During the three video game conditions, the lights in the room were turned off so that the display screen was clearly visible. A reflective marker was placed on the heel of each shoe at the ankle height just prior to game play and a lamp with light directed at the participants feet was used to facilitate visibility of the reflective marker used for step counting in the video recording.

Stepping and marching repetitions were extracted from the videos for all conditions. Two assessors (one had acquired the videos, the other verified the counts) manually counted the steps. A protocol for acceptable counts was developed and validated. Inter-rater reliability was established. Step repetitions were determined by the number of steps completed for each 30-s period defined by limb and direction of step. For example, if the participant was instructed to step with their right foot forward for 30 s, the number of steps with that extremity in that direction for 30 s was the frequency. The total number of steps over each 8.5-min bout was also considered when extracting and analyzing frequency.

Accuracy of the VREP, VRAN, and Kinect-LR stepping was extracted from the videos. Two assessors manually counted the accurate steps. A step was considered accurate if the participant’s foot landed partially or fully within the target tile. Similarly, when recorded by the system, a step was considered accurate if the participant stepped into the correct tile as required by the game. The foot was considered “on target” when half of the foot had entered the target tile. Within the game, this is ensured using object colliders both on the foot (placed around the arch of the foot) and the tile (around the boundaries). Only counts from the video data were included in the analysis. The SOC did not have an accuracy requirement.

#### Cardiovascular intensity: heart rate and metabolic equivalents

Raw data from the Cosmed system were exported as a Microsoft Excel file. In Excel, the data were visually inspected to verify the markers for the start and end time of each bout. Any erroneous markers were removed, and the bouts were labeled. In Matlab (MathWorks, United States), a three-minute plateau was selected and filtered using a Butterworth bandpass (30–200 Hz) filter. The plateau was defined as stable data extracted 240 s from the end of the exercise condition. The volume of oxygen consumption (VO_2_), percent of maximum heart rate (%HR_max:_: % (220-age)) and metabolic equivalent (METS = (VO2/Kg)/ 3.5) were calculated using standard formulas [44].

#### Perceived exertion, enjoyment, and activity preference rankings

Perceived exertion measured with the RPE scores for the mid-point and end of each condition were tallied. Enjoyment data were scored with the PACES. The eleven negatively worded items were reversed and summed with the scores for the positively worded items, resulting in a total score. Enjoyment was also was also analyzed with five-item version mPACES that focuses on game play [25]. The test scored items 1, 3, 6, 10 and 12 for a total of 35 points. They were expressed as a percent. The preference rankings were tallied. Open ended questions were reviewed for themes that explain the numerical data.

### Data analysis

The mean values (steps and march repetition, %HR_max_ and METs), maximum METs, and steps accuracy were calculated for each subject and then used for analysis on the group level. Box plots were drawn to display variation in samples (whiskers denote range, box denotes interquartile range, center line denotes median). Data were assessed for equal variance using the Mauchly sphericity test. Where the sphericity could not be assumed the Greenhouse–Geisser correction was used. Repeated measures ANOVA and post hoc paired t-tests with Holm's/Sidak correction for the number (2 or 3) of comparisons were calculated to test for differences in repetitions and accuracy (total steps, steps of the affected and unaffected lower extremities, total march repetitions, step accuracy of each limb), heart rate and metabolic equivalent (%HRmax, METs), and perceived exertion and enjoyment (RPE scores, PACES and mPACES scores expressed as a %, ranking preference) between the study conditions. Level of significance was set on value alpha ≤ 0.05 with corrections for comparison as noted above. For the measures of perceived exertion and enjoyment, minimal clinically important difference (MCID) was estimated with the distribution method by taking half of the baseline SD of the score [45] in each study condition. In analyzing differences between the pairs, the highest MCID value calculated for each pair was used as the MCID limit value for the results interpretation. Microsoft Excel 2010 (Microsoft Corp., Redmond, WA, USA, 2010) and IBM SPSS Statistics 26 (IBM Corp., Armonk, NY, USA, 2019) were used to calculate descriptive statistics, perform statistical tests and produce graphs.

## Results

### Participants

The study included 15 participants. Their age ranged from 38 to 72 years. The mean time post-stroke was 8 years. They presented with mild to moderate severity on the lower extremity section of the Fugl-Meyer Assessment Scale (FMAS) [40, 46, 47]. The characteristics of the participants are summarized in Table 2. One subject did not complete all four conditions of the study. Therefore, his data were excluded from the statistical analysis; data of 14 subjects were analyzed (n = 14).

**Table 2:** Participant’s characteristics, use of walking aid, and FMAS score (n = 15)

Characteristic	Value
Gender (n = male/female)	10 / 5
Age (years, mean ± SD)	55.4 ± 14.3
Time post stroke (years, mean ± SD)	8.3 ± 7.6
Hemiparetic side (n = right/left)	9 / 6
Walking aid (n)	
In the community	11
During training conditions	8
Orthotic (n)	9
FMA (score, mean ± SD)	21.4 ± 4.67

Values are reported as mean ± standard deviation unless otherwise indicated

FMA—the Lower extremity section of the Fugl-Meyer Assessment Scale

### Neuromuscular intensity and accuracy

**Fig. 2:**
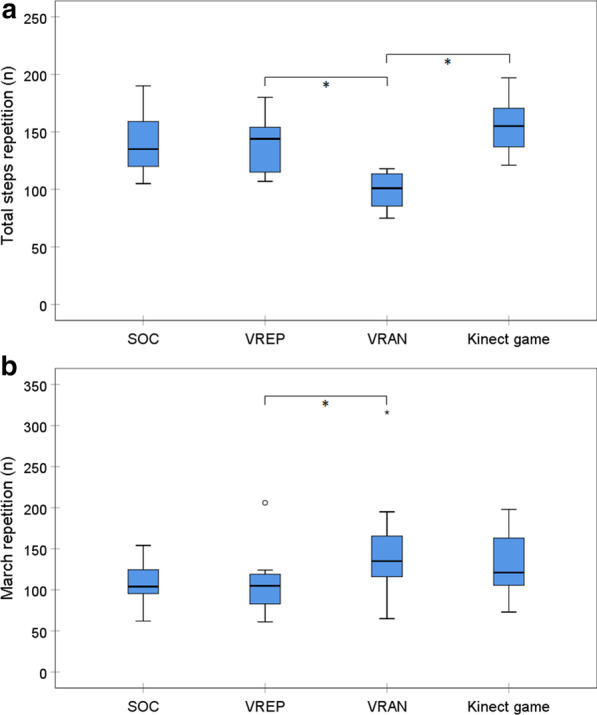
Comparison of total steps repetition (**a**) and total march repetition (**b**) between standard of care (SOC) and three active video games (n = 11)

Neuromuscular intensity measured by number of repetitions differed significantly between the four study conditions for total steps repetitions (F_(3)_ = 13.252; p < 0.001) and for march repetitions (F_(3)_ = 4.669; p = 0.009). The post hoc analyses showed significant differences between VREP and VRAN. For VREP, the number of total steps repetition was higher (t_(10)_ = 8.748; p < 0.001), but march repetition was lower (t_(10)_ = − 3.358; p = 0.011) (see Fig. 2a and b). Total step repetitions were significantly higher for Kinect-LR than VRAN (t_(10)_ = 6.700; p < 0.001). The difference in march repetition between these two conditions was not statistically significant.

The same pattern of differences was found also with analysis of the steps repetition by limb. The repetitions of the affected (F_(3)_ = 11.500; p < 0.001) and the unaffected (F_(3)_ = 9.029; p < 0.001) lower extremities differed significantly between the four study conditions. The post hoc analyses showed significantly higher number of steps repetitions for VREP than VRAN for both the affected (t_(10)_ = 4.345; p = 0.001) and the unaffected (t_(10)_ = 4.992; p = 0.001) lower extremities. Also, the steps repetition of each limb was significantly higher for Kinect-LR than VRAN (affected: t_(10)_ = 6.013; p < 0.001 and unaffected: t_(10)_ = 5.895; p < 0.001). There was no significant difference between SOC and VREP for any of the repetition parameters.

**Fig. 3:**
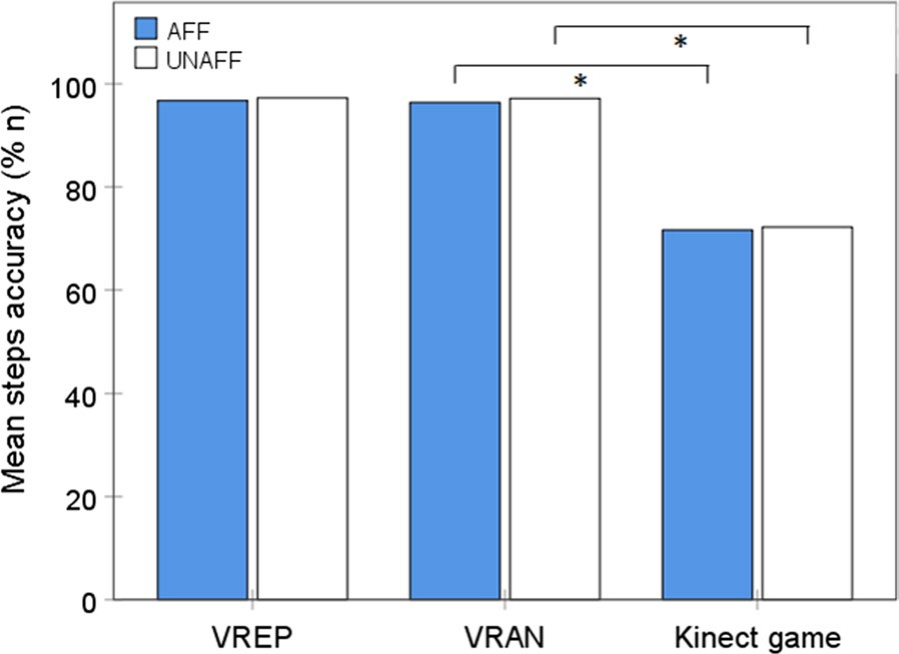
Comparison of step accuracy with the affected (AFF) and the unaffected (UNAFF) lower extremities between three active video games (n = 9)

Steps accuracy of the affected (F_(1.03)_ = 10.614; p = 0.011) and the unaffected (F_(1.03)_ = 17.680; p = 0.003) lower extremities differed significantly between the three virtual reality conditions. The post hoc analyses showed significantly better steps accuracy for VRAN than Kinect-LR for both the affected (t_(8)_ = − 3.418; p = 0.009) and the unaffected (t_(8)_ = − 4.577; p = 0.002) lower extremities. There was no statistically significant difference in steps accuracy between VREP and VRAN, although the mean values were the highest for VREP (Fig. 3).

### Cardiovascular intensity: heart rate and metabolic equivalent

**Fig. 4:**
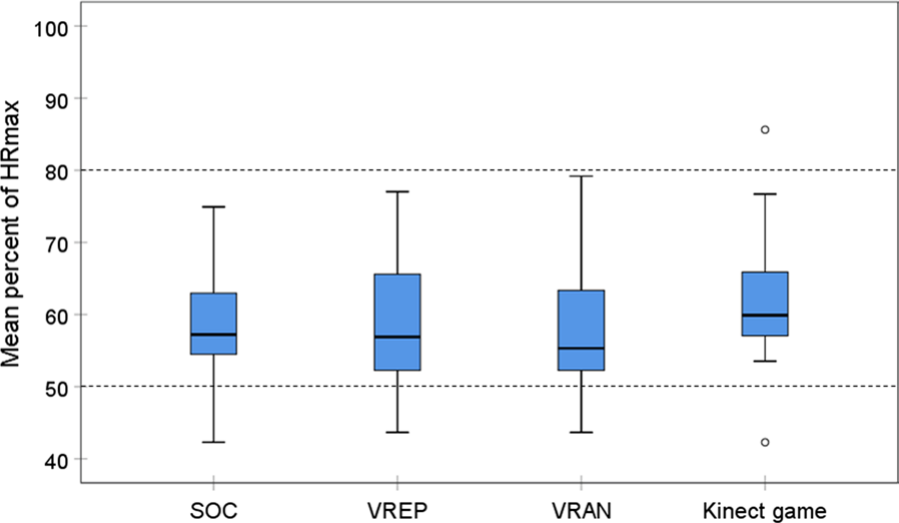
Comparison of mean percent of max heart rate between standard of care (SOC) and three active video games (n = 11). Area of recommended target heart rate for aerobic training for people after stroke is indicated between the doted lines [48]

Exercise intensity was measured by HR_max_ and energy expenditure (METs). There was no significant difference in mean %HR_max_ between the four study conditions. During all the conditions, exercise intensity was in the band recommended for aerobic exercise for people post stroke (Fig. 4).

**Fig. 5:**
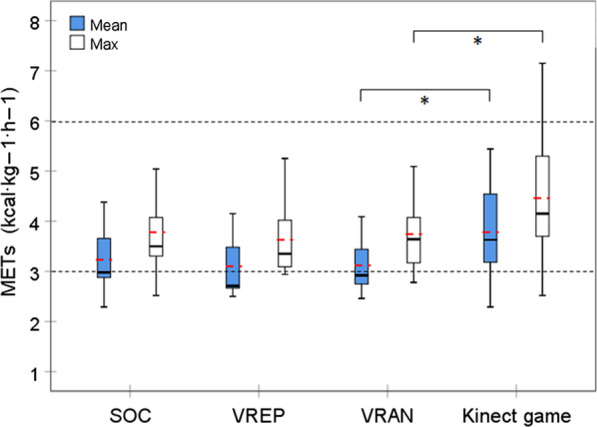
Comparison of mean and maximum metabolic equivalent (METs) between standard of care (SOC) and three active video games (n = 11). The mean values are marked with red dashed line. Area of moderate exercise intensity is indicated between the doted lines

The mean METs (F_(3)_ = 9.710; p < 0.001) and the maximum METs (F_(3)_ = 5.354; p = 0.004) differed significantly between the four study conditions. The post hoc analyses showed significantly greater values of both parameters for Kinect-LR than VRAN (mean METs: t_(10)_ = 3.803; p = 0.005 and max METs: t_(10)_ = 2.763; p = 0.030) and no significant difference between VREP and VRAN, and SOC and VREP. During all the conditions, the mean values of both METs parameters were in the band considered moderate exercise (Fig. 5).

### Perception of exertion and enjoyment

The RPE score differed significantly between the four study conditions (Table 3). The post hoc analyses showed significantly greater RPE score for SOC than VREP and for Kinect-LR than VRAN. There was no significant difference between VREP and VRAN.

**Table 3:** Comparisons of perceived exertion, enjoyment and ranking of preference between the study conditions

	Condition	Mean ± SD	RMANOVA		t-test with post hoc correction			MCID
			F	p value	Comparisons	t	p value	
RPE (n = 14)	SOC	14.93 ± 1.98	_(df: 1.74)_ 3.887	0.041	SOC–VREP	2.795	0.023*	1.08^#^
	VREP	13.71 ± 2.16			VREP–VRAN	1.223	0.122	1.56
	VRAN	12.79 ± 3.12			Kinect LR–VRAN	2.197	0.047*	1.56
	Kinect-LR	13.64 ± 3.13						
PACES (n = 14)	SOC	79.71 ± 23.70	_(df: 3)_ 2.824	0.051	SOC–VREP	− 2.080	0.058	11.85
	VREP	85.79 ± 19.55			VREP–VRAN	-1.547	0.073	9.77
	VRAN	90.57 ± 16.64			Kinect LR–VRAN	− 2.454	0.043*	9.61
	Kinect-LR	81.07 ± 19.22						
mPACES % (n = 14)	SOC	65.10 ± 20.59	_(df: 2.18)_ 3.349	0.046	SOC–VREP	− 1.458	0.084	10.3
	VREP	69.59 ± 13.38			VREP–VRAN	− 2.406	0.032*	7.51
	VRAN	76.12 ± 15.01			Kinect LR–VRAN	− 2.587	0.034*	8.84^#^
	Kinect-LR	64.09 ± 17.68						
Ranking preference (n = 12)	SOC	3.25 ± 1.14	_(df: 3)_ 4.591	0.009	SOC–VREP	3.386	0.009*	0.57^#^
	VREP	2.08 ± 0.51			VREP–VRAN	1.173	0.133	0.43
	VRAN	1.75 ± 0.87			Kinect LR–VRAN	2.382	0.036*	0.62^#^
	Kinect-LR	2.92 ± 1.24						

*RPE* Borg rating scale of perceived exertion, *PACES* physical activity enjoyment scale, *SOC* standard of care, *df* degrees of freedom, Post hoc Holm's/Sidak correction for 3 comparisons; *comparison between the two conditions p < 0.05; ^#^difference between conditions is clinically important > MCID

Similarly, participants’ ranking of game preference was significantly different. They liked the SOC condition the least, which was significantly lower than VREP, and they liked Kinect significantly less than VRAN (Table 3). Enjoyment measured by the full PACES scale was on the borderline statistically significant (p = 0.01). Exploratory post hoc analysis showed significantly greater enjoyment for VRAN than the Kinect only (Table 3). In contrast the short version of the PACES showed a significant difference between conditions with the VRAN preferred over the Kinect-LR and VREP.

## Discussion

Cardiovascular intensity based on METs and HR was comparable for all conditions. While there were statistically significant differences favoring the off-the-shelf Kinect-LR game for the METs, all conditions were in the band associated with moderate exercise intensity which meet the recommended guidelines for promotion of PA for persons post-stroke [48]. In contrast, movement intensity based on step repetitions was found to be greater for Kinect-LR stepping gameplay and both the marching and stepping gameplay for the custom self-paced VREP game. Therefore, it appears that similar neuromuscular intensity (repetitions) was achieved by either a custom AVG or the off-the-shelf AVG. Importantly, the intensity of the off-the-shelf Kinect-LR game was achieved at the expense of movement accuracy when compared to both custom AVGs. This speed accuracy trade-off may be important if movement accuracy as well as kinematics are a consideration when including AVGs in rehabilitation of persons post-stroke.

AVGs have an advantage over SOC as they use visual feedback facilitating an external focus of attention and can visually represent knowledge of performance (KP) and knowledge of results (KR). According to the OPTIMAL theory, these attributes can contribute to enhanced motor performance and motivation [49]. Persons post-stroke have demonstrated that they can use visual feedback KP from force generated through the lower extremities to reduce LE asymmetry and improve walking [50], as well as increase the paretic ground reaction force of the stroke affected lower extremity [51], and from electromyography selectively recruit lower extremity muscles during cycling task [52]. Combining visual KP and KR is easily achieved with virtual reality and has been shown across studies to improve balance for persons post-stroke in the both the subacute [53] and chronic phase [14, 54]. The off-the-shelf AVGs can provide the external focus of attention and KR for motivation, but they lack the specificity of movement tracking and therefore cannot offer KP or promote sound kinematics. Previously we reported that kinematics of stepping differed for the VREP compared to SOC. Specifically showing that the lack of visual feedback (SOC) resulted in a reduced step height for the affected lower extremity producing a march height asymmetry. Further, side stepping was also asymmetrical for the SOC and not for the VREP [35]. The cardiovascular intensity findings combined with movement accuracy findings recommend the custom games over both the standard of care activities and off-the shelf AVGs for persons post-stroke.

Consistent with our hypothesis, enjoyment was greater and perceived rate of exertion was lower for the custom AVGs. Specifically, the game-paced random (VRAN) game was rated as most enjoyable with the questionnaire (PACES) and confirmed with the preference rankings. Similarly, perceived exertion was lowest for the game-paced random (VRAN) game. Enjoyment using the PACES was previously reported for a customized game for upper limb rehabilitation post-stroke [55]. The magnitude of their score (65.8) is considerably smaller than that reported here. Enjoyment of AVGs played by persons post-stroke using the PS2 and Wii consoles [5, 29] and in particular the Kinect video games (both game-paced sports and the self-paced 20,000 leaks) has also been previously reported. [11] Persons post-stroke who used video games in community-based program reported that they experienced video games as a motivation tool rather than treatment [56]. We extend the findings reported by others about video game enjoyment by to comparing enjoyment and perceived effort between custom AVGs, standard of care and off-the-shelf AVGs.

Comparison between the customized games that were either self-paced (VREP) or game-paced (VRAN) yielded interesting results. Neuromuscular intensity was found to be greater for the VREP for steps but the opposite for marches. We speculate that the lower complexity of the VREP where movements are predictable and repeated made it easier to have more steps. The marching difference may be explained by conservation of energy, whereby the VRAN condition was less fatiguing during stepping (with lower repetitions) and was able to exert more effort during the marches. Cardiovascular intensity was comparable between the two custom game conditions. Ratings of the custom games for enjoyment and perceived effort were comparable. This finding was surprising as we anticipated that the random game would be preferred because of the variety. A possible explanation for this unexpected finding is that regardless of game mode participants prefer games that are within their physical abilities. This was possible by customizing the stepping volume for each participant. This is consistent of what persons post-stroke report they want in AVGs [32]. It is also in line with recommended principles of active video game design that suggest games are to be client-centered [31, 57]. Further, enjoyment is an important consideration for training outcomes and has been shown (also measured with the PACES) to be positively related to improved upper extremity performance in persons post-stroke [55]. The custom game preference was validated with both statistical significance and clinically important differences.

The most interesting finding of this study is that benefits of customization appear to be game mode neutral. Customization of games offers several advantages. Among them is the ability to address multiple therapeutic goals with the same game. The fact that there is no difference in enjoyment or perceived effort and both custom games (either self or game paced) were played in the moderate exercise band [48] suggests that either of the custom games could be used to promote PA. Enjoyment is a positive emotion linked to intrinsic motivation [58] and is both a predictor and an outcome of PA participation [59, 60]. It is an individual’s perception related to competence and personal preference, which can be associated with the type of PA, intensity level, environmental conditions, competition, and whether the activity takes place in an individual or group format [58]. Due to importance of PA for adults, enjoyment is an important construct for understanding PA participation, so the anticipated physical and psychological benefits can be realized. It may be possible to incorporate the AVGs designed for the VSTEP both for PA promotion as well as addressing balance and mobility goals in therapy.

Selecting AVGs for practice has several considerations including, customization, engagement and promotion of therapeutic goals. Lohse and colleagues [61] proposed six key factors to evaluate video games: reward, the right difficulty/challenge balance, performance feedback, choice and interactivity, clear goals and mechanics as well as socialization. In this study we focused on the balance of difficulty and challenge, performance feedback as well as clear goals and mechanics. By customizing the volume of stepping, we were able to balance the difficulty/challenge that in turn allowed for positive feedback on performance while not sacrificing intensity. Ultimately, achieving the exercise intensity that has been shown to be important for cardiovascular, neuromuscular and neural plasticity [62], while preserving movement control, as well aligning with patient’s preferences, may recommend custom games as reliable tool for both the promotion of PA and movement re-education.

Findings of the study should be interpreted with some caution as they are based on a small sample. The findings are reported based on playing the games during only a single session. The participants had mild-moderate lower extremity deficits but were able to do repeated stepping using their assistive device and brace, but did not require external support. Further while some of the findings are statistically significant not all of them are clinically meaningful. Neuromuscular intensity counted with stepping repetitions does not have reference values to interpret their clinical meaning. For enjoyment, perceived effort and ranking preference, a statistical method was used to interpret clinical meaning as there was also no benchmark for a reference. The explicit link between enjoyment and motivation was not measured and remains an important area of study [63].

## Conclusion

Custom AVGs that were either self or game paced were shown to promote comparable neuromuscular (repetitions) and cardiovascular (HR max and METs) intensity to carefully matched off-the-shelf AVGs and standard of care activities while resulting in more accurate movements. They were also reported to be more enjoyable and perceived to be less effortful. The custom games provided similar outcomes regardless of whether they were played in a self-paced or game paced mode. These findings add to the body of knowledge about the value of customization in game design for rehabilitation of persons with mild to moderate lower extremity deficits post-stroke.

## Data Availability

Yes.
